# NMR-Based Μetabolomics of the Lipid Fraction of Organic and Conventional Bovine Milk

**DOI:** 10.3390/molecules24061067

**Published:** 2019-03-18

**Authors:** Constantinos G. Tsiafoulis, Christina Papaemmanouil, Dimitrios Alivertis, Ouranios Tzamaloukas, Despoina Miltiadou, Stéphane Balayssac, Myriam Malet-Martino, Ioannis P. Gerothanassis

**Affiliations:** 1 NMR Center, University of Ioannina, GR-45110 Ioannina, Greece; 2 Laboratory of Analytical Chemistry, Department of Chemistry, University of Ioannina, GR-45110 Ioannina, Greece; 3 Section of Organic Chemistry and Biochemistry, Department of Chemistry, University of Ioannina, GR-45110 Ioannina, Greece; christina.pa@hotmail.gr; 4 Department of Biological Applications and Technology, University of Ioannina, GR-45110 Ioannina, Greece; dimitris.alivertis@gmail.com; 5 Department of Agricultural Sciences, Biotechnology and Food Sciences, Cyprus University of Technology, P. O. Box 50329, Limassol 3603, Cyprus; ouranios.tzamaloukas@cut.ac.cy (O.T.); despoina.miltiadou@cut.ac.cy (D.M.); 6 Biomedical NMR Group, SPCMIB Laboratory, Université Paul Sabatier, 118 route de Narbonne, 31062 Toulouse CEDEX, France; balayssac@chimie.ups-tlse.fr (S.B.); martino@chimie.ups-tlse.fr (M.M.-M.)

**Keywords:** metabolic profile, NMR, organic milk, CLA, UFA, 1D TOCSY

## Abstract

Origin and quality identification in dairy products is an important issue and also an extremely challenging and complex experimental procedure. The objective of the present work was to compare the metabolite profile of the lipid fraction of organic and conventional bovine milk using NMR metabolomics analysis. ^1^H-NMR and 1D TOCSY NMR methods of analysis were performed on extracted lipid fraction of lyophilized milk. For this purpose, 14 organic and 16 conventional retail milk samples were collected monthly, and 64 bulk-tank (58 conventional and 6 organics) milk samples were collected over a 14-month longitudinal study in Cyprus. Data were treated with multivariate methods (PCA, PLS-DA). Minor components were identified and quantified, and modification of the currently used equations is proposed. A significantly increased % content of conjugated (9-*cis*, 11-*trans*)18:2 linoleic acid (CLA), α-linolenic acid, linoleic acid, allylic protons and total unsaturated fatty acids (UFA) and decreased % content for caproleic acid were observed in the organic samples compared to the conventional ones. The present work confirms that lipid profile is affected by contrasting management system (organic vs. conventional), and supports the potential of NMR-based metabolomics for the rapid analysis and authentication of the milk from its lipid profile.

## 1. Introduction

Origin and quality identification in dairy products is an important issue within the food sector and is of major concern for consumers, retailers, food processors and regulatory authorities [1]. Milk is a complex biological fluid which contains water, lipids, carbohydrates, proteins, vitamins, minerals and various small molecular weight metabolites [2]. The metabolite composition of milk changes with feeding, management practices, stage of lactation, season, breed and health status of the animals [3,4,5,6,7]. Research on milk and dairy products has highlighted the benefits of their consumption to gastrointestinal health and immune system [8,9]. Milk and dairy product fat, which is rich in saturated fatty acids (SFA), has been linked to increased risk of cardiovascular disease-based primarily on the serum LDL-cholesterol raising effect. However, recent clinical and meta-analysis studies have shown that milk fat may be beneficial with regard to mortality, ischemic heart disease, stroke and diabetes [10], while other studies have related dairy fat with reduced risk of cardiovascular disease [11], diminished weight [12] and reduced risk of colorectal cancer [13].

Within the fast growth and expansion of the organic food market, the organic dairy market is increasing mainly due to consumer perception that organic milk is better than conventional and may also have potential benefits to human health. Milk fatty acid composition has been a central research area when comparing organic and conventional milk due to the fact that milk fatty acid profile is very sensitive to changes in diet of the animals [14,15]. It was suggested that approximately 50% of bovine milk fat is synthesized from plasma lipids, of which 88% are of dietary origin [16]. A key question, therefore, is whether the fatty acid profile of milk obtained from either organic dairies or from food markets is different from the milk either from conventional dairies or from food markets, e.g., rich in unsaturated fats, conjugated linoleic acids (CLAs) or ω-3 fatty acid content, which are important for cell membrane function, and have beneficial effects (such as with respect to cardiovascular disease, infant development, etc.) [17,18,19].

Accurate chemical analysis of lipids is important for determining the metabolite profile and nutritive value of organic dairy products and in preparing nutritional labeling of products for a particular function or application. The analysis of lipids, however, is extremely challenging and complex [20], since it can be very time-consuming and laborious and may require various preparation and analysis steps [21]. Methods widely used include gas chromatography (GC), GC-mass spectrometry (GC-MS), GC-tandem mass spectrometry (MS/MS), LC-UV, liquid chromatography- mass spectrometry (LC-MS), and LC-MS/MS [22,23]. However, GC methods involve several manipulation and derivatization steps that may cause oxidation of lipids [24,25], as well as undesirable isomerization processes [26]. NMR-based metabolomics has been widely used across several disciplines, including nutrition research [27]. NMR-based foodomics [28,29] has been successfully applied to investigate variations in the milk metabolite profile [30,31,32,33]. These studies are summarized in recent review articles [5,34]. Nevertheless, metabolomics investigation on milk and dairy product lipid fractions is limited to the ^1^H-NMR characterization of cow and buffalo milk [35], ^13^C-NMR investigation of milk from different animal species [36] and ^1^H and ^13^C-NMR identification of the production chain of *Asiago d’Allevo* cheese [37]. Recently, the combined chemometric analysis based on ^1^H-NMR, ^13^C-NMR and stable isotope data for the differentiation of organic and conventional milk has been reported [38].

The aim of the present study was to provide an NMR targeted and non-targeted metabolomics analysis for the rapid identification and quality determination of the lipid fraction of organic and conventional bovine milk with particular emphasis on metabolites of potential beneficial effects on human health, i.e., conjugated linolenic acids (CLAs), α-linolenic acid, linoleic acid and unsaturated fatty acids (UFAs). Taking into consideration the aims, constraints and limitations of high-throughput screening, a simple lipid fraction was used, and spectra were acquired with a high-resolution medium-field NMR spectrometer.

## 2. Results and Discussion

### 2.1. Spectral Analysis of Minor Components Using 1D ^1^H and 1D ^1^H TOCSY NMR

**Figure 1:**
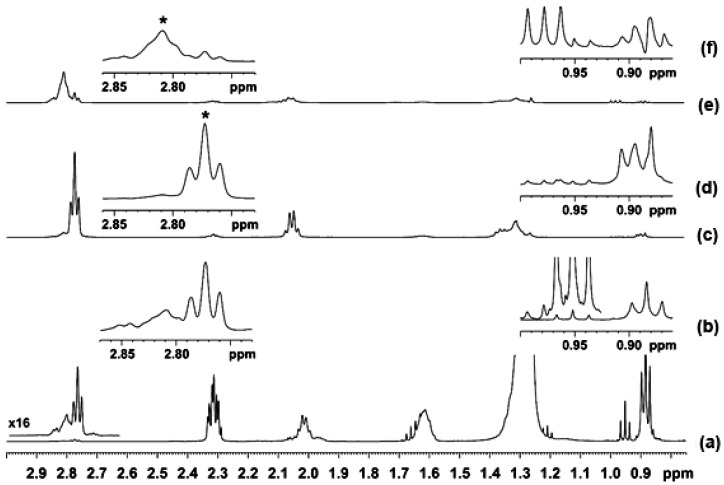
(**a**) Selected region of a 500 MHz ^1^H-NMR spectrum of the lipid fraction of a lyophilized bovine milk sample in CDCl_3_. (**b**) Magnification of the respective ^1^H-NMR regions T= 298 K; number of scans, 256; acquisition time 4.3 s; relaxation delay, 5 s; total experimental time ~25 min). (**c**) and (**e**) 1D TOCSY spectra; (**d**) and (**f**) are the magnification of the respective ^1^H-NMR regions, τ_m_ = 200 ms, number of scans, 256 and total experimental time ~25 min; the asterisks (*) denote the resonances of the allylic protons that were excited with the use of a selective shaped pulse of 80 ms.

**Scheme 1:**
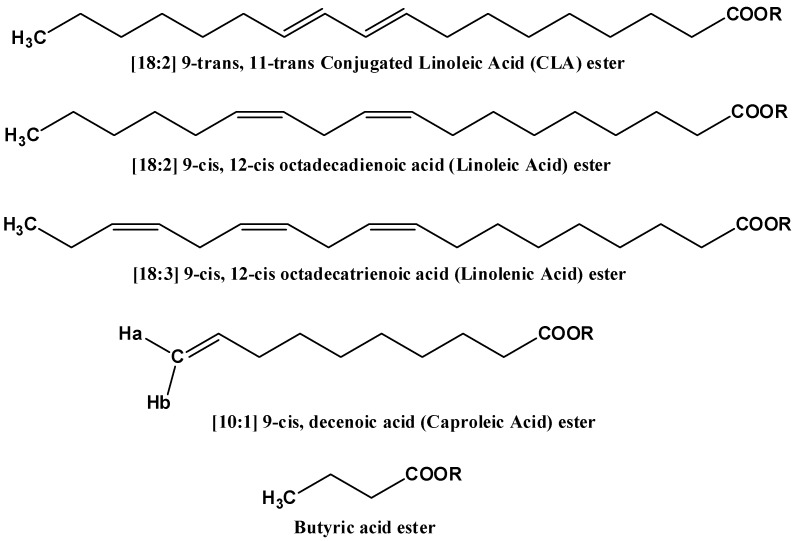
Chemical structures of the compounds investigated in the present metabolomics study.

**Figure 2:**
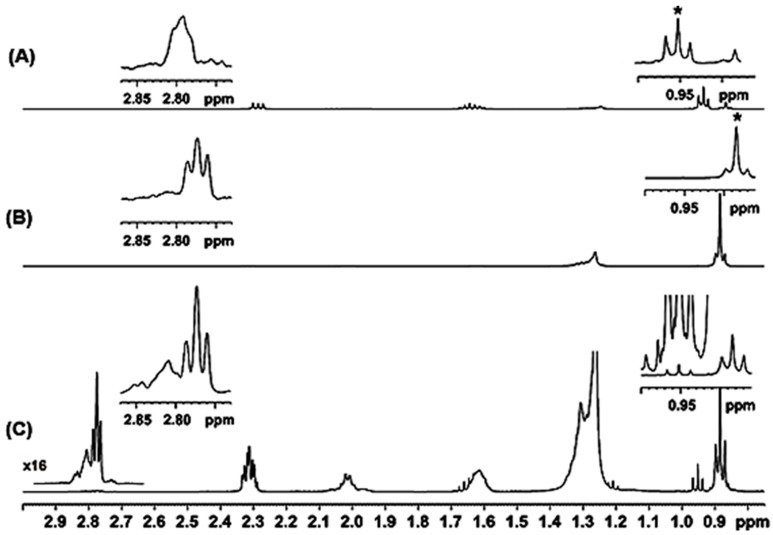
(**A**) Selected region of a 500 MHz ^1^H-NMR spectrum of the lipid fraction of a lyophilized bovine milk sample in CDCl_3_; T, 298 K; number of scans, 256; acquisition time, 4.3 s; relaxation delay, 5 s; total experimental time ~25 min. (**B**) and (**C**) 1D TOCSY spectra with τ_m_ = 200 ms, number of scans, 256 and total experimental time ~25 min. The asterisks (*) denote the –CH_3_ resonances that were excited with the use of a selective shaped pulse of 80 ms.

**Figure 3:**
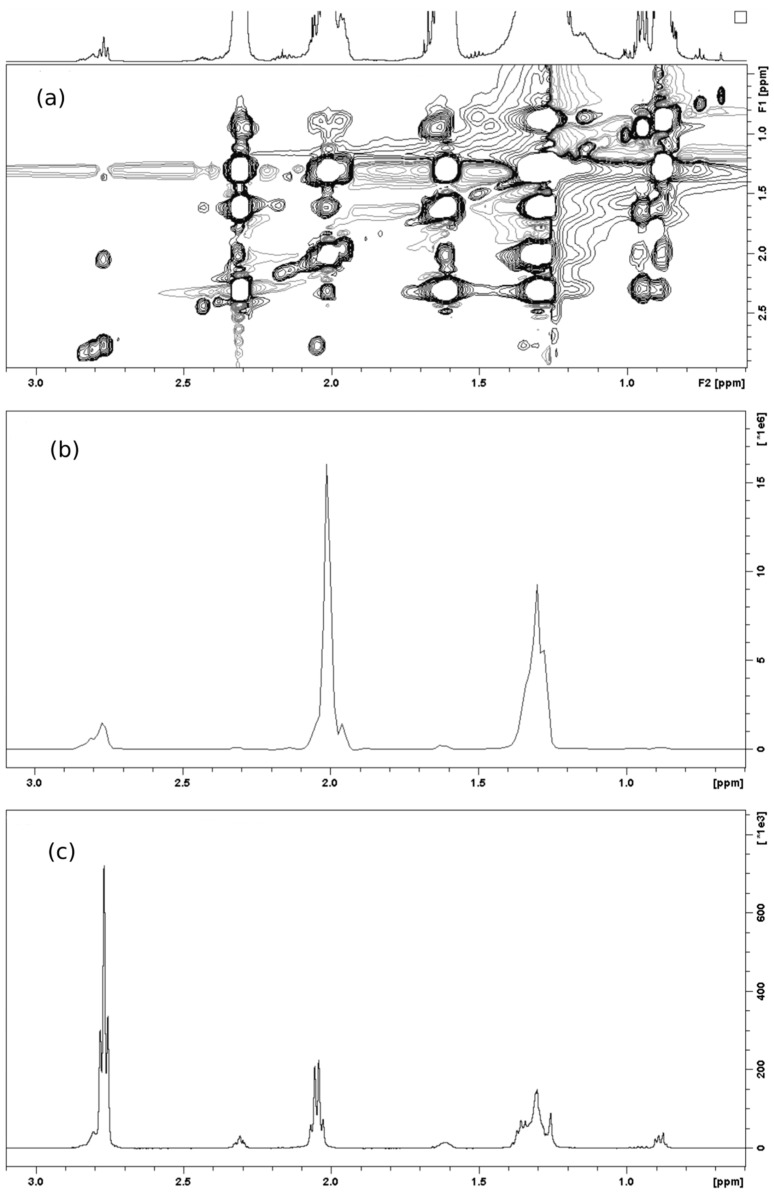
(**a**) Selected region of 500 MHz 2D TOCSY spectrum of the lipid fraction of a lyophilized bovine milk sample in CDCl_3_ (T = 298 K, 32 repetitions of 512 increments, total experimental time 5 h 5 min); (**b**) selected row on the bis-allylic protons of linoleic acid (δ = 2.77 ppm) of the 2D TOCSY spectrum as displayed in (**a)**; (**c**) 1D TOCSY of the same solution as in (**a**) with the selective pulse on the bis-allylic protons at δ = 2.77 ppm is illustrated for comparative reasons (number of scans, 256; acquisition time 4.3 s; relaxation delay, 5 s; total experimental time ~25 min).

The ^1^H-NMR spectrum of the lipid fraction of lyophilized milk sample illustrates the major signals due to the protons of the triacylglycerols (TAG) at 4.15 ppm (*sn*-1 and *sn*-3 H_a_ protons), 4.28 ppm (*sn*-3 and *sn*-1 H_b_ protons) and 5.27 ppm (*sn*-2), the olefinic protons –CH=CH– at ~5.33 ppm, the protons of 1,2 diacylglycerols (DAG) (3’–CH_2_–OH) at 3.73 ppm, the protons –C(2)H_2_–COOR at ~2.33 ppm, –CH_2_–CH=CH at ~2.02 ppm, –CH_2_–CH_2_–COO(R) at ~1.62 ppm, –(CH_2_)_n_– at ~1.32 ppm and 1.27 ppm, the –CH_3_ protons of butyric acid at 0.95 ppm, and the –CH_3_ of fatty acids at ~0.88 ppm [35,39]. An exemplary collection of spectra is shown in Figure 1. The signals at 2.77 to 2.81 ppm which appear at higher (×16) vertical magnification can be attributed to bis-allylic protons from the –CH_2_– groups located between pairs of double bond and, thus, provide a measure of the number of poly unsaturated ω-3 and ω-6 fatty acids present in the sample. The bis-allylic protons of α-linolenic acid (Scheme 1) are more deshielded, δ = 2.81 ppm, than in linoleic acid (δ = 2.77 ppm) presumably due to a larger number of double bonds in the former case (Figure 1a). Furthermore, α-linolenic acid (18:3 ω-3) contains a double bond close to the terminal –CH_3_ group that is known to cause a shift to higher ppm values from 0.88 to 0.99 ppm. Selective 1D TOCSY excitation with 200 ms mixing time of the bis-allylic protons at 2.81 ppm (Figure 1e) demonstrates the effective magnetization transfer to H18 methyl group at 0.98 ppm, although this group was completely hidden in a conventional 1D ^1^H-NMR spectrum under the resonance of the –CH_3_ group of the abundant butyric acid (δ = 0.95 ppm) with ~10^2^ stronger signal intensity. Similar results were obtained with selective 1D TOCSY excitation of the –CH_3_ groups at 0.88 and 0.95 ppm which demonstrate the effective magnetization transfer to the bis-allylic protons at 2.77 ppm and 2.81 ppm, respectively (Figure 2). The above NMR chemical shift data are in excellent agreement with the Livestock Metabolome Database (LMDB), which provides additional confirmation of the assignement of linoleic and α-linolenic fatty acids. Figure 3 illustrates a comparison of the 1D TOCSY spectrum of a lyophilized bovine milk sample with the respective row in the 2D TOCSY experiment. The standard 2D TOCSY experiment requires ~5 h acquisition time to achieve a comparable sensitivity to the 1D TOCSY experiment that is obtained in only ~25 min. It can be concluded that in metabolomic studies requiring a large number of samples and with particular emphasis on a limited number of metabolites of potential beneficial effects on human health, the 1D TOCSY allows for the unequivocal assignment and structure elucidation of minor components in a time-efficient manner, with excellent spectral resolution, and without t1 noise artifacts that affect the analytical performance of 2D NMR experiments (Figure 3a).

**Figure 4:**
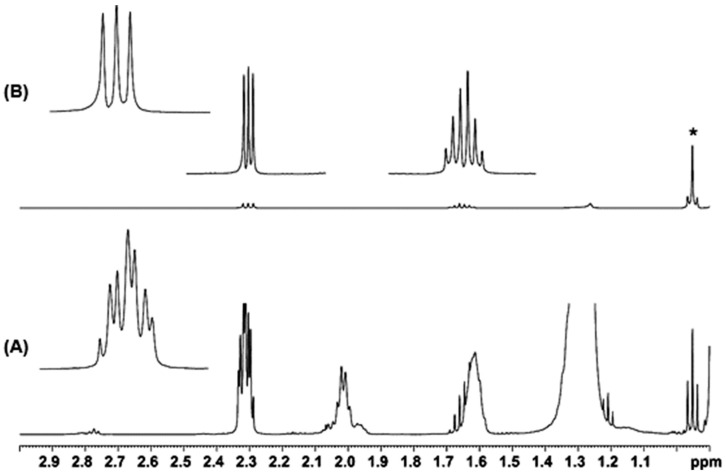
(**A**) Selected region of a 500 MHz ^1^H-NMR spectrum of the lipid fraction of a lyophilized bovine milk sample in CDCl_3_; T, 298 K; number of scans, 256; acquisition time, 4.3 s; relaxation delay, 5 s; total experimental time ~25 min. (**B**) 1D TOCSY spectrum with τ_m_ = 200 ms, number of scans, 256 and total experimental time ~25 min. The asterisks (*) denotes the –CH_3_ group of butyric acid that was excited with the use of a selective shaped pulse of 80 ms.

Selective 1D TOCSY excitation of the –CH_3_ group of butyric acid at 0.95 ppm with τ_m_ = 200 ms resulted in the effective magnetization transfer throughout the complete proton spin system at 1.65 and 2.32 ppm, although the C(2)H_2_COOR protons were completely hidden in the conventional 1D ^1^H-NMR spectrum (Figure 4). These chemical shift values are in excellent agreement with the NMR data for butyric acid of the LMDB. Interestingly, the C(2)H_2_COOR protons of butyric acid appear as a distorted triplet (δ = 2.32 ppm, ^3^*J* = 7.2 Hz) because of the coupling with the C(3)H_2_ protons, while the C(2)H_2_COOR protons of the major lipids at δ = 2.31 ppm exhibit a complex multiplet pattern. Fatty acids are not randomly esterified at the three positions of the TAG molecule. The short-chain acids, such as butyric acid (4:0) and caproic acid (6:0), are esterified almost entirely at the *sn*-3 position and only one short chain can occur per TAG molecule. Medium-chain fatty acids (8:0–14:0) as well as 16:0 are preferentially esterified at positions *sn*-1 and *sn*-2. Stearic acid (18:0) is selectively placed at position *sn*-1, whereas oleic acid (18:1) shows preference for positions *sn*-1 and *sn*-3 [40]. The triplet of the –C(2)H_2_COOR protons of butyric acid (Figure 4) clearly demonstrates the presence of only one short chain at *sn*-3 position of the TAG molecule in excellent agreement with literature data ([34] and references herein). It remains to be seen whether the 1D TOCSY experiment could provide a novel method in estimating the distribution of medium and long chain fatty acids within the TAG molecule.

**Figure 5:**
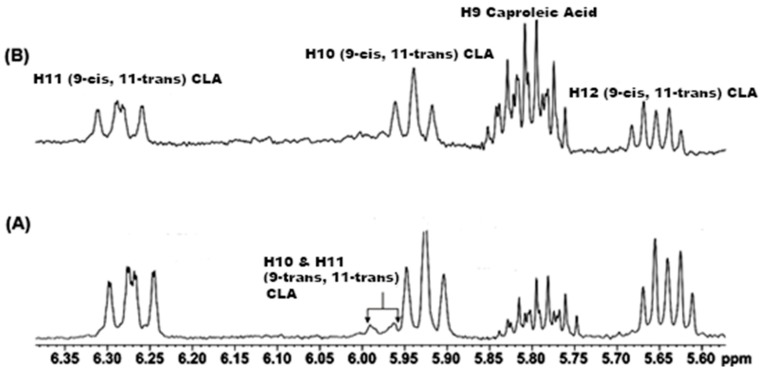
Selected region of 500 MHz ^1^H-NMR spectra of the lipid fraction of lyophilized organic (**A**) and conventional (**B**) bovine milk samples in CDCl_3_ (T, 298 K; number of scans, 256; acquisition time 4.3 s; relaxation delay, 5 s; total experimental time ~45 min). Some characteristic resonances from CLA and caproleic fatty acids are indicated.

Figure 5 illustrates the region of the olefinic protons of CLAs. The resonance at 6.28 ppm has been attributed to the signal of H11 of the (9-*cis*, 11-*trans*) 18:2 CLA isomer [37,39,40,41,42]. The contribution of the H11 resonance of the (10-*trans*, 12-*cis*) 18:2 CLA positional isomer at δ = 6.29 ppm [43] and of the H10 resonance of the (9-*trans*, 11-*cis*) 18:2 CLA isomer at 6.28 ppm [44] was found to be of minor importance in all milk samples examined. The weak resonance at 5.99 ppm was attributed to the H10 and H11 protons of the (9-*trans*, 11-*trans*) 18:2 CLA geometric isomer [41,44]. Further confirmation of the above assignment was achieved with the use of ^1^H-^13^C HSQC NMR experiments (Figure S1), The H11 at 6.28 ppm correlates with the C11 at 125,58 ppm, the H10 at 5.93 ppm correlates with the H10 at 128.65 ppm and H12 at 5.64 ppm with C12 at 134.71 ppm, in agreement with literature data [44]. Unfortunately, the LMDB does not include NMR chemical shift data of CLAs.

### 2.2. Quantification Using 1D NMR

Fatty acid composition of the milk was determined by calculating the integrals, *I*, of specific NMR signals following the method of Brescia et al. [35]. Evaluation of the linoleic and α-linolenic acid concentration was based on the integrals of the signals due to bis-allylic moieties at 2.77 ppm (*I*_2.77_) and 2.81 ppm (*I_2_*_.81_) using the following equations:

(1) $$\alpha -\mathrm{linolenic}\;\mathrm{acid}\;(\%)=\frac{3I_{2.81}}{2\left(I_{0.95}+I_{0.88}\right)}\frac{3I_{2.81}}{4\left(I_{0.95}+I_{0.88}\right)}=\frac{3I_{1.8}}{4I_{TL}}$$

(2) $$\mathrm{linoleic}\;\mathrm{acid}\;(\%)=\frac{3\;I_{2.77}}{2\left(I_{0.95}+I_{0.88}\right)}=\frac{3I_{2.77}}{2I_{TL}}$$

where *I_TL_* is the sum of the total integrals (*I*_0.95_ + *I*_0.88_), where *I_0_*_.95_ corresponds to the –CH_3_ group of α-linolenic acid and butyric acid at 0.98 and 0.95 ppm, respectively, and *I_0_*_.88_ the integral of all the –CH_3_ groups in the region of 0.88 ppm.

Evaluation of the total (9-*cis*, 11-*trans*) 18:2 CLA, (9-*trans*, 11-*cis*) 18:2 CLA and (10-*trans*, 12-*cis*) 18:2 CLA concentration was based on the integrals of the signals at 6.28 ppm, 6.28 ppm and 6.29 ppm that correspond to H11, H10 and H11 protons, respectively, *I_6_*_.28_; evaluation of the (9-*trans*, 11-*trans*) 18:2 CLA concentration was based on the integral of the signals due to H10 and H11 protons at 5.99 ppm, *I*_5.99_, using the following equations:

(3) $$\begin{array}{c} {}[(9-cis,\;11-trans)+(9-trans,\;11-cis)+(10-trans,\;12-cis)]\;18:2\;\mathrm{CLA}\;(\%)=\frac{3\;I_{6.28}}{I_{TL}} \end{array}$$

and

(4) $$(9-trans,\;11-trans)\;18:2\;\mathrm{CLA}\;(\%)=\frac{3\;I_{5.99}}{2I_{TL}}$$

Evaluation of the caproleic acid concentration was based either on the integral of the H9 proton at 5.80 ppm (*I*_5.80_) or of the H10a proton at 4.99 ppm (*I*_4.99_) using the following equation:

(5) $$\mathrm{Caproleic}\;\mathrm{acid}\;(\%)=\frac{3I_{5.80}}{I_{TL}}=\frac{3\;I_{4.99}}{I_{TL}}$$

The percentage of monounsaturated fatty acids (MUFA) can be obtained by subtracting the percentages of linoleic, *α*-linolenic and 18:2 CLA fatty acids from the total amount of UFAs, using a partially modified equation of Brescia et al. [35] as follows:

(6) $$\begin{array}{c} \mathrm{UFA}(\%)=\mathrm{MUFA}+\mathrm{linoleic}\;\mathrm{acid}+\alpha -\mathrm{linolenic}\;\mathrm{acid}+18:2\;\mathrm{CLA}=\frac{\frac{D-2I_{4.99}}{4}}{\frac{C-I_{4.99}}{2}}=\frac{1}{2}\frac{D+2I_{4.99}}{C+I_{4.99}} \end{array}$$

where *D* is the integral of the CH_2_–CH=CH protons at 2.02 ppm (*I*_2.02_) and *C* is the integral of the C(2)H_2_COOR protons at 2.33 ppm (*I*_2.33_). The modified term in Equation (6) was introduced in order to take into consideration the concentration of caproleic acid which is the only MUFA with a terminal double bond, and is resonating in the *I*_2.02_ and *I*_2.33_ regions.

SFA content can be obtained by subtracting the UFA content from the total fatty acids (TFA) as follows:

(7) $$\mathrm{SFA}\;(\%)=\frac{I_{0.88}}{I_{TL}}+\frac{I_{0.95}}{I_{TL}}-\frac{1}{2}\frac{D+2I_{4.99}}{C+I_{4.99}}$$

### 2.3. Analytical Performance Characteristics

#### 2.3.1. Robustness

Extraction experiments were performed at 25 °C, 30 °C and 35 °C to investigate the effect of temperature. The % relative deviations at 30 °C and 35 °C, with respect to those obtained for caproleic acid at 25 °C, were found to be −2% and −5%, respectively. The effect of pH was also investigated at pH = 6.8 (natural pH value of the milk sample), 6.5 and 7.1. The % relative deviations for caproleic acid at pH = 7.2 and 6.5, with respect to pH = 6.8, were found to be −4.2 and −6.1%, respectively. These experiments demonstrate the robustness of the method in the temperature range of 25 to 35 °C and at pH = 6.8 ± 0.4.

#### 2.3.2. Precision

Precision can be expressed by the coefficient of variation (CV or % rsd) [45], which was calculated for three samples of the same origin and taking three ^1^H-NMR measurements per sample. The CV was calculated to be 1.5%, thus demonstrating the repeatability of the method. The precision of the analytical procedure was calculated as prec(χ) = 1 − rsd (χ) = 0.985. This demonstrates the high precision of the method and its effectiveness in the analysis, on a statistical basis, of a large number of samples.

### 2.4. Metabolomic Study of Organic and Conventional Milk Samples

#### 2.4.1. Classification of Milk Samples

Metabolomic study of the ^1^H-NMR spectra of the lipid fraction of 94 samples (30 P and 64 R samples, see also Section 3.1) was performed in order to investigate the origin of the samples (organic (O) or conventional (C)). The milk samples were divided in two major subgroups: “raw” samples, R, which refer to untreated milk samples that were collected from the bulk-tank, of each farm, and “processed” samples, P, that were collected from commercial packaging. Clustering of the data was visualized either through score plots, in which each point represents an individual sample, or through loading plots which resample the original spectrum, and thus permit the identification of the most important spectral regions and individual metabolites which are responsible for each clustering.

As previously described, for the bucketing of the recorded ^1^H-NMR spectra, area values were used unmodified (UNor), since normalization to the total spectra is discouraged [46]. For a second data series, values were normalized (NorCont) in order to take into consideration the deviations in the lipid content of each sample; prior to the multivariate analysis, each bucket was multiplied by a factor of three and then was divided by the sum of the signal buckets that refer to the total sum of areas of the –CH_3_ signals (peaks at 0.88 ppm, *I*_0.88_, and at 0.95 ppm, *I*_0.95_). The main advantage of the above procedure is that each analyte could be expressed as % content in the lipid fraction of each sample (Equation (8)).

(8) $$\frac{\left(\mathrm{Integral}\;\mathrm{of}\;\mathrm{the}\;\mathrm{compound}\right)\times 3}{\left(\mathrm{number}\;\mathrm{of}\;\mathrm{protons}\;\mathrm{of}\;\mathrm{compound}\right)\times I_{TL}}$$

Thus, following the general hypothesis of one proton per each bucket, the “normalized” buckets that were calculated were further subjected to multivariate analysis.

Following the above-mentioned procedures, two data series were obtained: UNor and NorCont. For the bin areas scaling of the two data series, the UNor and the NorCont, Pareto scaling was used [47]. This procedure reduces the relative importance of large variances but keeps data close to their original values. Moreover, it reduces the mask effect from abundant metabolites (whereas, for instance, UV scaling magnifies the variations due to low-abundance metabolites) [19].

**Figure 6:**
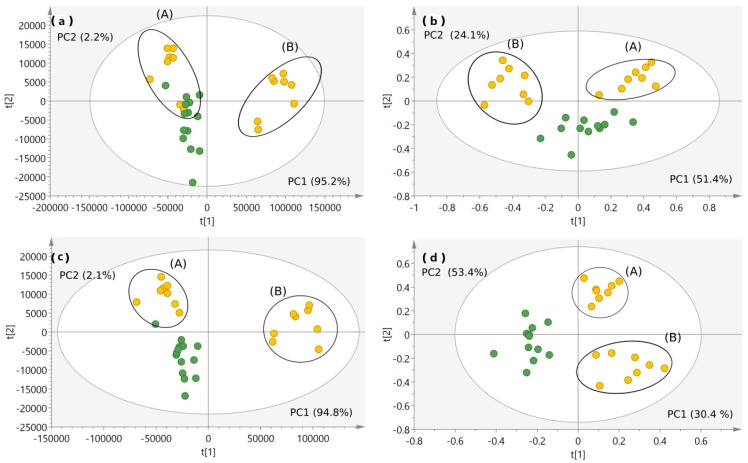
Score plots obtained after the PCA and PLS-DA analyses on the UNor and NorCont data of the lipid fraction of organic and conventional processed (P) milk samples. (**a**) PCA score plot of UNor procedure, (**b**) PLS-DA score plot of the NorCont procedure, (**c**) PCA score plot of the UNor procedure, (**d**) PLS-DA score plot of the NorCont procedure. Yellow circles for conventional and green circles for organic samples; (A), conventional milk samples consisted of 1.5% fat content and (B), conventional milk samples consisted of 3% fat content.

PCA and PLS-DA analyses were performed. Samples were classified into two groups with respect to the sampling method (P or R). Figure 6 shows the PCA and PLS-DA score plots for the two methods for the P samples and the PC1 and PC2 percent values. The UNor procedure (PC1 and PC2 in PCA analysis accounted for 97.4% of the total variance) shows, for the conventional samples (CP), separation due to different fat content for (A) and (B) subgroups corresponding to fat content of 1.5% and 3%, respectively. Using the NorCont procedure, two outliers were revealed and excluded from the multivariate analysis, and PC1 and PC2 in PCA analysis accounted for 75.5% of the total variance (Figure 6c). Interestingly, despite the fact that the PC1 in the UNor method accounted for 95.2% of the total variance (Figure 6a), whereas with the NorCont method this was ~51.4% for the PC1 and 24.1% for the PC2 (Figure 6c), the “elimination” of the different fat content, which also results in an increase in the PC2 contribution to the total variance is displayed in the PLS-DA score plot (Figure 6d). In this plot, we have group variations within, but not between, the two subgroups. This could be explained by the fact that for every sample, the lipid content is “normalized” since we refer to % content instead of areas (see discussion above). This is of importance since we could study the populations without the impact of the difference in the lipid content between the samples.

In the PLS-DA analysis of the P samples, following the UNor procedure, the PC1 and PC2 accounted for 96.9% of the total variance, and analysis showed good group clustering (R^2^Y value was 0.80) and class discrimination (Q^2^ value of 0.70). The NorCont procedure (PC1 and PC2 accounted for 83.8% of the total variance) showed similar good group clustering and class discrimination (R^2^Y value was 0.67 and Q^2^ was 0.76). The PLS-DA analyses of the R samples, following the UNor procedure, were 0.61 and 0.44 for R^2^Y and Q^2^, respectively, having poor predictive capability. NorCont revealed 5 outliers that were excluded from further analysis, and showed similarly to the UNor procedure group clustering and class discrimination (the obtained values of R^2^Y and Q^2^ were 0.47 and 0.30, respectively). This is expected, since the “normalization” method that was applied in the NorCont procedure reduced the inhomogeneity of the samples, due to the different lipid content of each sample; this results in similar subgroup clustering and class discrimination for the two procedures.

The permutation test validation and internal cross-validation, based on their respective p-value of the Fischer’s exact test, were also evaluated. For the P group, the predictive capability through internal cross validation showed a *p*-value of the Fischer’s exact test equal to 6.9 × 10^−9^ (in both procedures) with 100% correct predictions. The PLS-DA validation, through permutation test using an internal cross-validation method (100 iterations), showed that NorCont and UNor models are valid. As shown in Figure S2, where all the permuted R^2^ and Q^2^ values to the left are lower than the original point to the right, the blue regression line of Q^2^ has a negative value (−0.46) for both NorCont and UNor. In the R samples, the inhomogeneity of the lipid content of the samples resulted in poorer Q^2^ values compared to the P samples for both procedures. Moreover, the NorCont procedure performed better than UNor, considering the above reported criteria. More specifically, the predictive capability through internal cross validation showed a *p*-value of the Fischer’s exact test equal to 1.6 × 10^−5^ and 95.0% correct predictions in UNor, whereas in the NorCont procedure the respective *p*-value was better (2.1 × 10^−6^), and 100% correct prediction was obtained. Both PLS-DA models were cross-validated through permutation analysis (*n* = 100), showing goodness of fit and prediction; they had lower R^2^Y values compared with the original model and negative intercept of the Q^2^ regression lines. Moreover, the validation of the PLS-DA through permutation test was slightly improved for the NorCont procedure compared to the UNor (Q^2^ value of −0.25 for NorCont, and −0.11 for UNor for the P samples).

To validate the models’ performance, R samples were used as a test set in the PLS-DA models. NorCont performed better in terms of predictive capabilities with respect to UNor, showing the applicability of the obtained model in class discrimination. As shown in Figure S3, the majority of the predicted samples were outside the 95% confidence level with UNor (all the O samples are outliers), whereas with NorCont, although several samples were outside the 95% confidence level, the method could correctly predict the two classes.

#### 2.4.2. Marker Identification and Quantification

PLS-DA was analyzed with respect to the impact of the analytes through the Variable Importance in the Projection (VIP). VIP plots were constructed and the contribution of compounds of interest were examined, based on their impact to the variation and the correlation in the data set. A strong influence was observed: (i) of the allylic protons –CH_2_–CH=CH– (1.93 to 2.09 ppm) and resonances in the region of 2.09 to 2.18 ppm, (ii) the olefinic protons (acyl chains) –CH=CH– (5.33 to 5.43 ppm), (iii) the bis-allylic protons of linoleic acid (2.77 ppm) and α-linolenic acid (2.81 ppm), (iv) the (9-*cis*, 11-*trans*) 18:2 CLA (6.28 ppm) and (v) the caproleic acid (5.80 ppm). Both UNor and NorCont showed similar influence of the above analytes in both P and R samples.

Table 1 displays the quantitative data for the above metabolites of the lipid fraction. Moreover, two sample *t*-tests, for the R and P groups were also performed and the respective *p*-values are also presented. As shown in Table 1, significant increased % content of (9-*cis*, 11-*trans*) 18:2 CLA, α-linolenic acid, linoleic acid, allylic protons and UFA and decreased % content for caproleic acid were observed in the organic samples compared to the conventional ones.

#### 2.4.3. Nutritive Value of Organic *Versus* Conventional Milk Regarding Their Lipid Composition

There is increasing evidence that ω-3 and 18:2 CLA fatty acids impact health benefits to the consumer [48,49,50,51], and that the dietary balance of ω-3 and ω-6 fatty acids is perhaps as important as the dietary proportions of monounsaturated, saturated and total fat [52,53]. There was a significantly higher % content of the major 18:2 CLA isomers in organic milk compared to conventional milk, with these differences maintained over the duration of the present study. This is in agreement with the work of Jahreis et al. [54] and Bergamo et al. [55], but contrasts with the study of Ellis et al. [56], who found no difference in the major 18:2 CLA content between organic and conventional milk produced on farms in England and Wales with similar feeding practices.

The ω-6:ω-3 fatty acid ratio was found to be slightly increased in organic milk (in the range 2.79 ± 0.24 to 3.02 ± 0.01) compared to conventional milk (2.64 ± 0.12 to 2.84 ± 0.05). The optimum ω-6:ω-3 ratio in the human diet is 2.3, since at this ratio, the conversion of α-linolenic to long chain ω-3 docosahexaenoic acid (DHA) is suggested to be maximized [57]. However, the optimal ω-6:ω-3 ratio depends on genetic factors and health conditions, and even a 4:1 ratio was found to have a positive effect on asthma patients [58] and decreased mortality in patients with previous myocardial infarction [59]. Several studies have concluded that a reduced ω-6:ω-3 ratio, compared with values of ~15 in several European countries [60] and 10 to 15 in U.S.A. [53,61] diets, during adulthood, will lower risks for, e.g., cardiovascular disease [52,62], metabolic syndrome and diabetes [63,64] and overweight [12].

Unsaturated fatty acid content was increased in organic milk (~28%) compared to conventional milk (~24%). The significantly higher values of 18:2 CLA and ω-3 and ω-6 fatty acids that were found in organic milk in the present study affect its nutritional value, since the latter are essential fatty acids, whereas the 18:2 CLA is regarded as nutritious bioactive fat due to several biological beneficial effects [17]. The differences found in the present study in milk lipid fractions could be explained by the contrasting feeding regimes applied in organic (forage-based) and conventional (concentrate-based) farming in Cyprus, which supports the claim of Schönfeldt et al. [65] for country-specific milk data. This may be overemphasized in Cyprus where, due to water scarcity, conventional farms are basing their feeding regimes on concentrates, in contrast to organic farms, which are forced by EU regulation to have at least 60% of the animals’ diet sourced from forages and silages, which increases the unsaturation of milk lipids [66].

## 3. Materials and Methods

### 3.1. Milk Sample Collection

Fourteen organic and sixteen conventional retail bovine milk samples (referred as “processed”, P, samples) were collected during September 2011 to September 2012 from the largest, and only, processor that produces fresh conventional and organic milk in Cyprus. Organic milk consisted of 1.5% fat content (semi skimmed) whereas for conventional milk, half of the samples consisted of 3% fat content, and the remaining eight samples were semi-skimmed. All the above samples were previously homogenized and pasteurized using high-temperature short-time method and collected every month from commercial packaging (high-density polyethylene bottles) before doorstep delivery to minimize storage time in retail outlets, while maintaining availability to consumers.

Furthermore, another set of 64 bulk-tank milk samples (referred as “raw”, R, samples) were also collected from 15 conventional farms that had been previously selected as representative of dairy farming in Cyprus, and all organic farms producing cow milk (two in total). As a standard practice, 4 samples from each farm were collected throughout the year and all samples, after collection, were transported in opaque cool boxes to the laboratory, where they were transferred into 30 mL sterile, screw-top plastic bottles and stored at −20 °C until chemical analysis was carried out. NMR analysis was performed on 30 processed (16 conventional and 14 organic samples) and 64 raw (58 conventional and 6 organic) milk samples.

### 3.2. Chemicals

Conjugated (9-*cis*, 11-*trans*) 18:2 linoleic acid, purity ≥ 96% (HPLC) and conjugated (9-*trans*, 11-*trans*) 18:2 linoleic acid, purity ≥ 98% (HPLC), were purchased from Fluka Chemie GmbH (Buchs, Switzerland). Caproleic acid, purity ≥ 96%, and hexamethylcyclotrisiloxane, as chemical shift reference, were purchased from Sigma-Aldrich Chemie GmbH (Taufkirchen, Germany). Chloroform and methanol (analytical grade) were obtained from Fisher Scientific UK (Loughborough, UK) and CDCl_3_ (99.8%) from Deutero (Kastellaun, Germany).

### 3.3. NMR Analysis

The lipid fractions of the milk samples were prepared using the Bligh and Dyer method for 300 mg of lyophilized milk, as previously described [44]. The extracted lipid fraction was dissolved in 0.59 mL of CDCl_3_ and 0.01 mL of a 17.08 mM hexamethylcyclotrisiloxane solution was added as an internal chemical shift reference (δ = 0.172 ppm). The solution was then transferred into a 5 mm NMR tube. NMR experiments were performed on a Bruker AV500 spectrometer (Bruker Biospin, Rheinstetten, Germany) at 298 K using the Topsin 2.1 suite. All 1D ^1^H-NMR spectra were collected using a 30° flip angle, a spectral width of 14 ppm with the same receiver gain value, a relaxation delay of 5 s, several transients of 512, and an acquisition time of 4.3 s. 64 K data points were collected and the FIDs were treated using a line broadening exponential function of 0.3 Hz. Phase adjustment and baseline correction between 0.03 and 6.36 ppm were carried out using Topspin 2.1 suite. Signal integration for lipid quantification was manually done and was the same for all spectra. For the identification of minor analytes 1D TOCSY experiments were carried out using a standard Bruker pulse program (seldigp). A shaped pulse length of either 20 ms or 80 ms was used for selective excitation, followed by either a MLEV-17 TOCSY spin-lock or by applying the DIPSI-2 pulse train and by incorporating a z-filter before acquisition [67] for the suppression of artifacts. The shaped pulse length of 80 ms resulted in significantly better selectivity of the excitation bandwidth of the spin chromatographic method (Figure S4), which allowed the selective excitation even in closely spaced resonances as in the case of the allylic protons of linoleic acid (δ = 2.77 ppm) and α-linolenic acid (δ = 2.81 ppm) (Scheme 1, Table 2). The spin-lock was adjusted to 7.1 KHz, corresponding to a low-power 90° pulse of 35 μs; this allows safe operation without problems of significant heating of the samples with spin-lock times, τ_m_, up to 400 ms [41]. 2D ^1^H-^1^H TOCSY, ^1^H-^13^C HSQC and ^1^H-^13^C HMBC NMR experiments were recorded using standard Bruker software.

Particular attention was paid to the interpulse time delay, since this will affect the accuracy of the ^1^H-NMR integrals. Therefore, detailed investigation of the longitudinal relaxation times T_1_ was performed with the inversion recovery method (Table 2). To obtain optimum accuracy for quantification and avoid differential saturation effects, the proton spins should fully relax between pulses, demanding recycle times [(acquisition time) + (relaxation delay)] of at least 5 × Τ_1_ (when a 90° pulse is applied), where Τ_1_ is the longitudinal relaxation time of the slowest relaxing nuclei. In our experiments, a recycle time of 9.3 s was used, which was sufficient to achieve, using a 30° pulse, the complete relaxation (~99.2%) of all nuclei according to the Ernst angle. Figure S5 demonstrates the consistency of the peak integrals for two different recycle times of 9.3 s and 14.5 s. It should be emphasized that the relaxation times of the model compounds are significantly longer than those in milk samples. For instance, caproleic acid C(10)Ha and Hb protons had T_1_ = 3.2 s in milk samples, whereas it was 4.2 s when the standard compound was measured. This may be attributed to the fact that the model compounds are in free acid form, and not in the form of triglyceride esters, which implies significantly different correlation times for molecular tumbling, and thus relaxation properties.

### 3.4. Multivariate Data Analysis

Amix 3.2 software (Bruker, Biospin, Germany) was used to segment the ^1^H-NMR spectra with a fixed-size bucketing of 0.01 ppm for the region between 0.03 to 6.34 ppm and the buckets were integrated. Area values were used unmodified (UNor), or “normalized” (NorCont) (see Section 2.4.1). All data matrices were imported to SIMCA-P software version 14.0 (Umetrics, Umea, Sweden) and preprocessed with Pareto scaling. Principal component analysis (PCA) and Partial Least Squares projections on latent structures (PLS-DA) were performed. All models were validated using the segment cross-validation method, Q^2^, R^2^Y, Fisher’s exact test, and percentage of correct prediction, wherein each segment represents the replicates of each sample. A permutation test was also performed.

## 4. Conclusions

In this work, we have demonstrated the potential of NMR-based metabolomics [68] of the lipid fraction of organic and conventional bovine milk, through a careful selection of the analytes to be investigated. The method might become of primary interest in milk and dairy products metabolomics since: (i) it is rapid, selective and non-destructive, (ii) it allows the chemical identification of minor components even in strongly overlapping spectral regions, (iii) it does not require derivatization steps, and (iv) it enables the quantification of analytes of interest simultaneously in a single experiment, in contrast with classical analytical methodologies. The LC-MS methodology, on the other hand, has significantly higher sensitivity and is capable of determining lipid classes, species, fatty acid composition and triglyceride TAG formulation [69,70]. Nevertheless, the NMR methodology, for the purposes of the present investigation, is adequately used, since in dairy studies it enables the appraisal of the authenticity and geographical origin of milk [35,38], assesses important milk-related technological parameters [33] or nutritional qualities [30,32], and can be used to test the effects of organic feeding on milk characteristics in animal studies.

Our results show the discrimination between organic and conventional milk produced in Cyprus, with differences mainly being assigned to specific fatty acids, with increased % contents of (9-*cis*, 11-*trans*) 18:2 CLA, linoleic and *α*-linolenic acids, compounds with allylic protons and unsaturated fatty acid, while there was a decreased content of caproleic acid in organic milk compared to conventional. These data demonstrate the improved nutritive value of organic milk regarding its lipid composition and show that there are significant possible opportunities to improve the fatty acid profile, and thus nutritional quality, of milk and dairy products, especially from certified organic farms. Further usage of NMR-based metabolomics methods for the investigation of the “organic effect” will enable the implementation of management practices that will improve milk fatty acid composition.

## Figures and Tables

**Table 1:** Mean % content ^a^ of the metabolites (indicators) in the lipid fraction of milk samples. The *p*-value of the *t*-test is reported ^b,c^.

Metabolite	OR Samples	OP Samples	CR Samples	CP Samples
(9-*cis*, 11-*trans*) 18:2 CLA ^d^	0.49 ± 0.06	0.51 ± 0.06	0.30 ± 0.08	0.31 ± 0.03
*p*-value ^b^			3.98 × 10^−9^	
*p*-value ^c^				2.56 × 10^−11^
Caproleic acid	0.25 ± 0.03	0.23 ± 0.02	0.32 ± 0.04	0.33 ± 0.03
*p*-value ^b^			3.47 × 10^−5^	
*p*-value ^c^				4.72 × 10^−12^
α-linolenic acid	0.92 ± 0.08	0.88 ± 0.08	0.65 ± 0.06	0.61 ± 0.05
*p*-value ^b^			4.32 × 10^−18^	
*p*-value ^c^				2.45 × 10^−12^
Linoleic acid	2.67 ± 0.35	2.66 ± 0.22	1.72 ± 0.24	1.73 ± 0.11
*p*-value ^b^			1.12 × 10^−15^	
*p*-value ^c^				9.23 × 10^−15^
–CH_2_–CH=CH–	54.71 ± 2.68	54.47 ± 2.47	46.79 ± 3.45	47.98 ± 1.96
*p*-value ^b^			9.38 × 10^−9^	
*p*-value ^c^				1.61 × 10^−9^
UFA	28.00 ± 1.19	28.07 ± 1.36	24.33 ± 1.75	24.32 ± 1.22
*p*-value ^b^			7.48 × 10^−8^	
*p*-value ^c^				1.30 × 10^−8^

^a^ Expressed as (mean value) ± SD. ^b^ Comparison between organic raw (OR) and conventional raw (CR) samples. ^c^ Comparison between organic processed (OP) and conventional processed (CP) a samples. ^d^ The contribution of the H10 resonance of the (10-*trans*, 12-*cis*) 18:2 CLA positional isomer at δ = 6.28 ppm [43] and of the H10 resonance of the (9-*trans*, 11-*cis*) 18:2 CLA isomer at 6.29 ppm [44] was found to be of minor importance.

**Table 2:** Selected NMR signals of the milk lipid fraction and their relaxation times, Τ_1_, which were used for the quantification of analytes in milk samples.

Protons	Chemical Shift [δ (ppm)]	Relaxation Time T_1_ (s)
CH(12)=CH(11)—CH(10)=CH(9) *^a^*	6.28	1.5
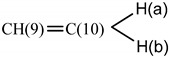	5.80 (H-9)	3.2
	4.99 (H-10) *^b^*	3.2
CH=CH–CH_2_–CH=CH *^c^*	2.81	1.5
CH=CH–CH_2_–CH=CH *^d^*	2.77	1.5
C(2)H_2_COOR	2.33	2.0
CH_2_–CH=CH–	2.02	2.0
CH_3_ *^e^*	0.95	1.7
CH_3_ *^f^*	0.88	1.7

*^a^* CH(11) of (9-*cis*, 11-*trans*) 18:2 CLA. *^b^* H(10a) of caproleic acid. *^c^* bis-allylic protons of α-linolenic acid. *^d^* bis-allylic protons of linoleic acid. *^e^* CH_3_ group of butyric acid. *^f^* CH_3_ group of fatty acids except ω-3 and butyric acid.

## Supplementary Materials

The following are available online. Figure S1: Selected region of 500 MHz ^1^H-^13^C HSQC spectrum. Figure S2: Permutation test of the P samples with PLS-DA. Figure S3: Predicted score plot of the R samples (as test set) using P samples as a prediction set. Figure S4: Selectivity of the excitation of the bandwidth of the shaped pulse. Figure S5: 500 MHz ^1^H-NMR spectra.
