# Supplementary material for: NMR-Based Μetabolomics of the Lipid Fraction of Organic and Conventional Bovine Milk
Source: Molecules. 2019 Mar 18;24(6):1067. doi: 10.3390/molecules24061067 (PMC6472053; doi:10.3390/molecules24061067)
Supplement: Supplementary file 1 [file molecules-24-01067-s001.pdf]

NMR-Based Metabolomics of the Lipid Fraction of Organic and Conventional Bovine Milk

Constantinos G. Tsiafoulis*[1,2], Christina Papaemmanouil[3], Dimitrios Alivertis[4], Ouranios Tzamaloukas[5], Despoina Miltiadou[5], Stéphane Balayssac[6], Myriam Malet-Martino[6], Ioannis P. Gerothanassis*[3]

[1] NMR Center, University of Ioannina, Ioannina GR-45110, Greece
University of Ioannina, Ioannina GR-45110, Greece
[2] Laboratory of Analytical Chemistry, Department of Chemistry; University of Ioannina, Ioannina GR-45110, Greece; University of Ioannina, Ioannina GR-45110, Greece
[3] Section of Organic Chemistry and Biochemistry, Department of Chemistry; University of Ioannina, Ioannina GR-45110, Greece; University of Ioannina, Ioannina GR-45110, Greece
[4] Department of Biological Applications and Technology;
University of Ioannina, Ioannina GR-451 10, Greece
[5] Department of Agricultural Sciences, Biotechnology and Food Sciences, Cyprus University of Technology, P. O. Box 50329, Limassol 3603, Cyprus
[6] Biomedical NMR Group, SPCMIB Laboratory, Université Paul Sabatier, 118 route de Narbonne, 31062 Toulouse cedex, France

* Corresponding Correspondence: authors: C. G. Tsiafoulis, e-mail: ctsiafou@uoi.gr (C.G.T.); I.P. Gerothanassis, email: igeroth@uoi.gr (I.P.G)

Formatted: English (United States)

Formatted: English (United States)

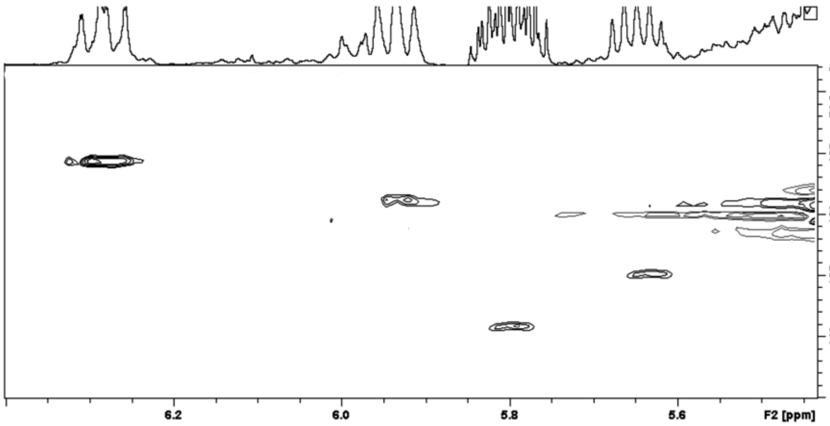

Figure S1. Selected region of 500 MHz ^{1}H-^{13}C HSQC spectrum of the lipid fraction of a lyophilized bovine milk sample in $CDCl_3$ illustrating ^{1}H-^{13}C connectivities of conjugated (9-*cis*,11-*trans*)18:2 linoleic acid (CLA) and caproleic acid; T, 298 K; 40 repetitions of 256 increments, total experimental time 4 h 35 min.

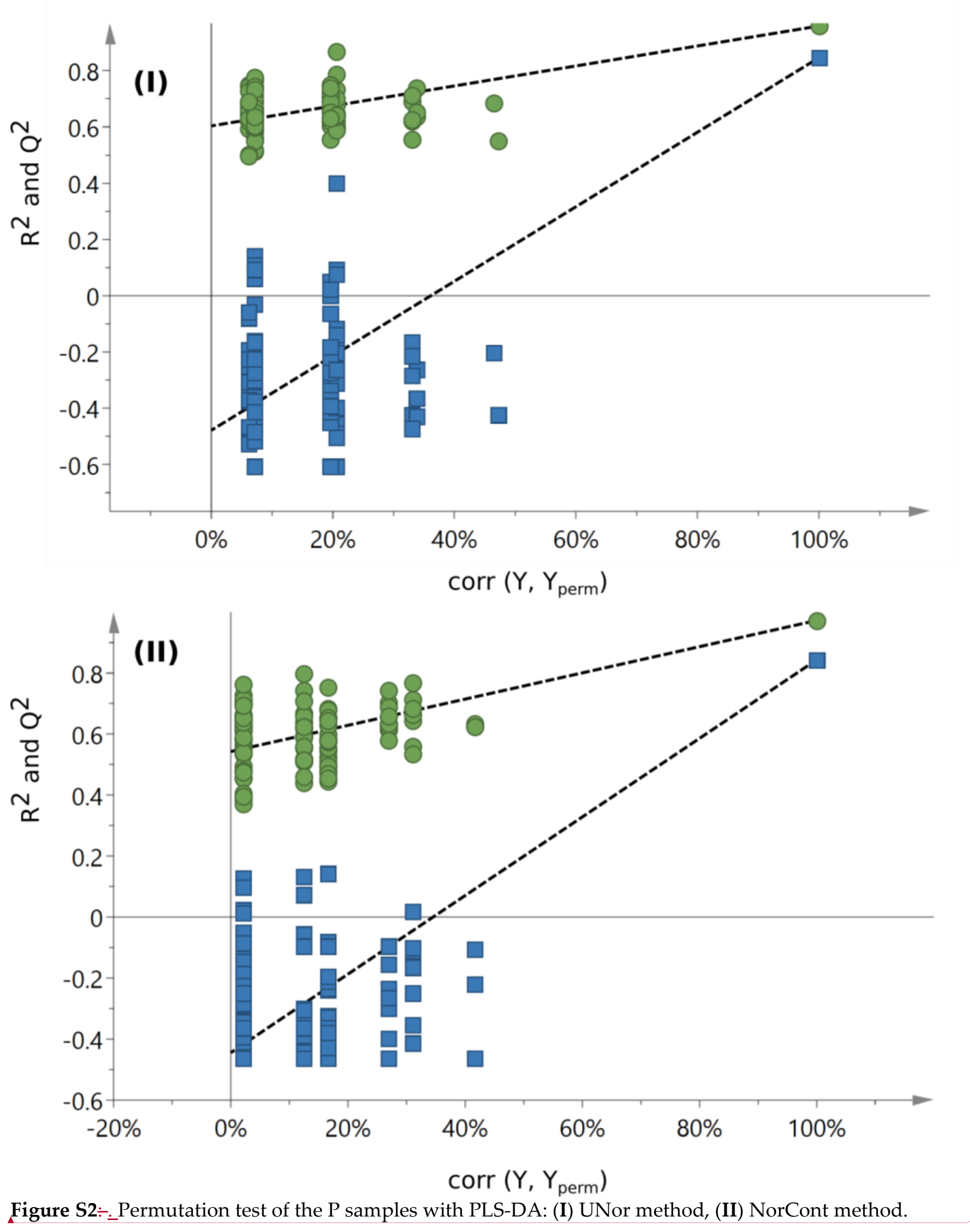

Figure S2. Permutation test of the P samples with PLS-DA: (**I**) UNor method, (**II**) NorCont method.

Formatted: Font: Bold

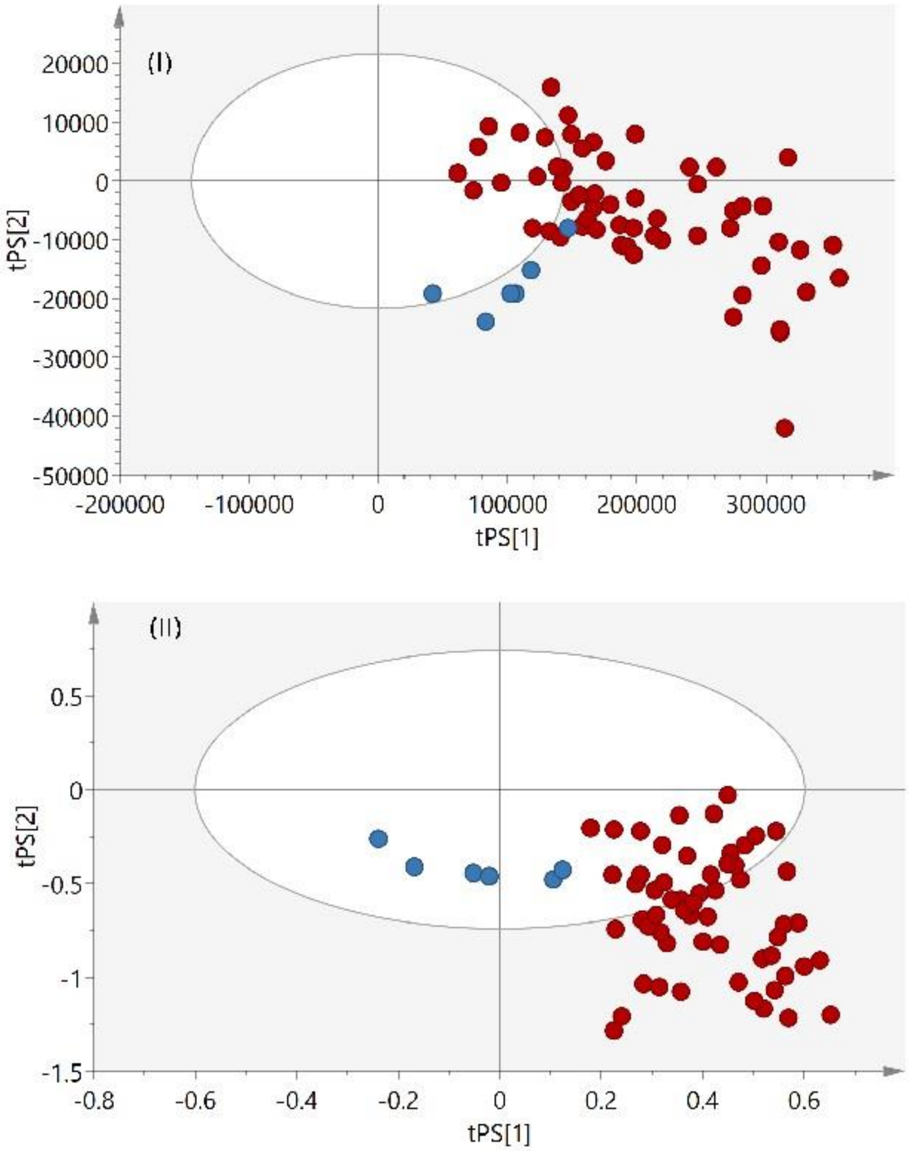

Figure S3. Predicted Score Plot of the R samples (as test set) using P samples as a prediction set: (**I**) UNor method, (**II**) NorCont method (blue circles: organic milk samples, red circles: conventional milk samples).

Formatted: Font: Bold

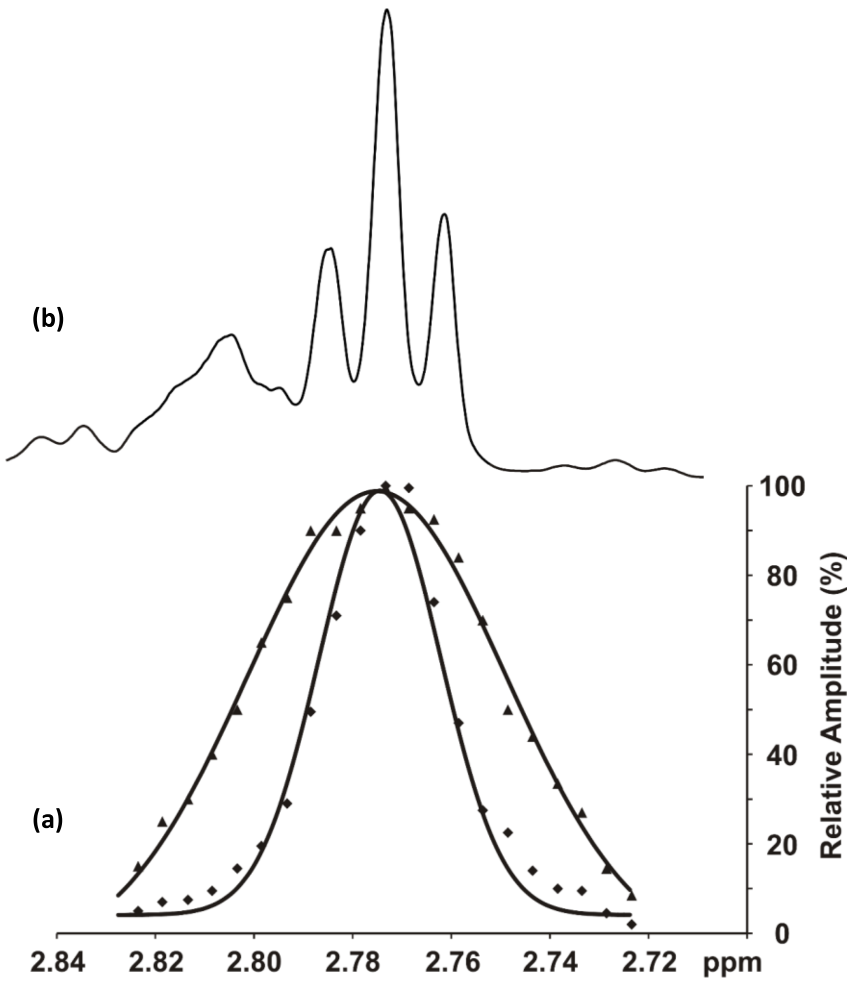

Figure S4. (**a**) Selectivity of the excitation bandwidth of the shaped pulse of 20 ms (▲) and 80 ms (♦) of the 1D TOCSY experiment. The carrier frequency of the shaped pulse was set in both cases at δ = 2.77 ppm. (**b**) For comparison the apparent triplets of the allylic protons of linoleic acid (δ = 2.77 ppm) and *α*-linolenic acid (δ = 2.81 ppm) of the 1D ^1H NMR spectrum of the lipid fraction of ~~lyoplilized~~ lyophilized bovine milk sample in $CDCl_3$ are presented.

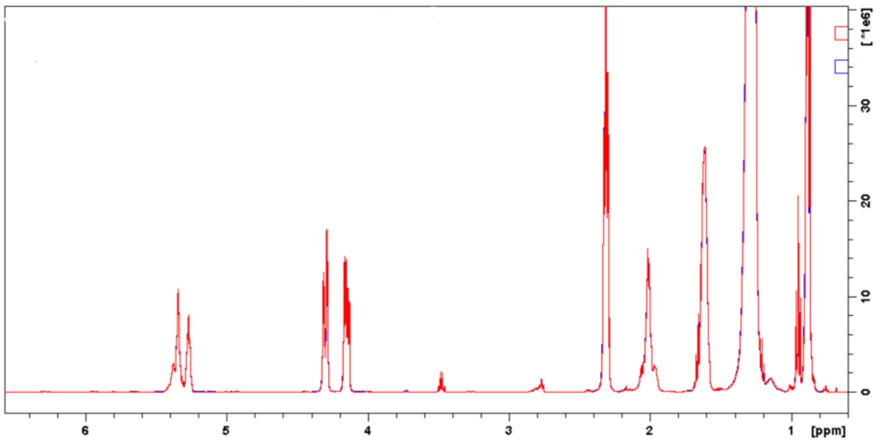

Figure S5. 500 MHz ^{1}H NMR spectra of the lipid fraction of a lyophilized bovine milk sample in $CDCl_3$; T, 298 K; Number number of scans, 256 ; recycle time, 9.3 s. (blue color) and 14.5 s (red color).
